# Editorial: Functional insights into the probiotic mechanisms of surface protein action

**DOI:** 10.3389/fmicb.2023.1355529

**Published:** 2024-01-05

**Authors:** Jai K. Kaushik, Vengadesan Krishnan, Ingemar von Ossowski

**Affiliations:** ^1^ICAR-National Dairy Research Institute, Karnal, India; ^2^Regional Centre for Biotechnology, NCR Biotech Science Cluster, Faridabad, India; ^3^Department of Veterinary Biosciences, University of Helsinki, Helsinki, Finland

**Keywords:** probiotics, surface proteins, molecular mechanisms, adhesion, host-microbe interactions, *Lacticaseibacillus rhamnosus*, *Limosilactobacillus reuteri*, *Lactiplantibacillus plantarum*

Many medical research and clinical studies have already documented the role played by probiotic bacteria in sustaining the overall intestinal health of humans and animals. On the other hand, there has been less revealed about the molecular mechanisms of the host-microbe interactions that enable these beneficial outcomes. Gut persistence and survival are often touted as important factors that typically dictate the endurance of the health-benefiting effects of a microbe in a host. Consequently, as the adhesion capacity of a probiotic strain has come to define its suitability and fitness for use, outer surface proteins and their interactions with host cell receptor sites, substrates, and other bacteria are an underlying facet of this functionality. However, for the most part, the detailed mechanisms of surface protein action still need to be explored and understood. This Research Topic has aimed to increase our knowledge about the way in which probiotics function and convey health benefits to hosts. Accordingly, we are delighted to have published five original research articles (El-Chami et al.; Pang et al.; Tian et al.; Muruaga et al.; Sha et al.) within this Research Topic. Collectively, these articles were contributed by 48 authors and add significantly to our understanding of the probiotic mechanisms of surface protein action and cascading effects by covering a wide range of relevant subject matter. Highlights of this five-article Research Topic are summarized below.

*Lacticaseibacillus rhamnosus* GG is a widely used probiotic whose cell lysate can protect human keratinocytes from the effects of the skin and mucosal pathogen *Staphylococcus aureus* by means of growth inhibition and competitive exclusion and displacement. In their study, El-Chami et al. speculated that this anti-adhesive activity results from quenched *S. aureus* receptor binding sites on keratinocytes via the selective adsorption of effector molecules present within the lysate fraction. The authors demonstrated that trypsin- or heat-treated cell lysates no longer displayed the protective role, thereby suggesting that the effector molecules are likely proteinaceous in nature. The authors went on to show that the SpaC pilus protein and specific moonlighting (or promiscuous) proteins are some of the lysate effector molecules that help inhibit staphylococcal-binding activity. Findings from this study deliver valuable and practical insights into a topical probiotic therapy for preventing and treating *S. aureus* skin infections and disease.

Pang et al. undertook to study the extracellular membrane vesicles (MVs) from two probiotically relevant strains (*Limosilactobacillus reuteri* DSM 17938 and BG-R46). In their study, Pang et al. examined the physicochemical, biological, and production properties of MVs from these two probiotic strains and found that MV production can be enhanced by prolonged cell growth and exposure to oxygen stress. Intriguingly, the authors also revealed that the *L. reuteri* MVs are comprised of numerous bacterial cell surface proteins, some being involved in host-bacteria interactions. To gauge possible host interactions, the authors scrutinized the efficacy of the MVs by using three different cell models. Here, MVs from *L. reuteri* were found to enhance epithelial barrier integrity, modulate cytokine production, and antagonize the pain receptor TRPV1.

Tian et al. investigated the control mechanisms that contravene oxidative (H_2_O_2_) stress in *Lactiplantibacillus plantarum* KM1, a strain isolated from natural fermented products with demonstrated probiotic and antioxidant properties, using proteomics. The authors showed that up to 112 differentially expressed proteins (DEPs) were identifiable (31 upregulated and 81 downregulated) under oxidative stress conditions. As a coping mechanism to the H_2_O_2_ stress, DEPs participating in various metabolic pathways (e.g., pyruvate, carbon, trichloroacetic acid cycle, amino acid, and DNA repair) were involved. Resulting from their proteomic analysis of the oxidative stress response in *L. plantarum* KM1, the authors suggested that a theoretical basis for the use of this strain as a natural antioxidant has been established.

S-layer proteins are known to self-assemble and form layered bi-dimensional lattices that cover bacterial cell surfaces, including those of probiotics like *Lactobacillus acidophilus*. Muruaga et al. developed a versatile tagging tool for recombinant protein purification by adapting the C-terminal domain from the *L. acidophilus* S-layer SlpA protein for use as a molecular tag. As this C-terminal region (coined by the authors as SLAP_TAG_) associates SlpA to the bacterial cell surface, it was used to develop the SLAP_TAG_-based affinity chromatography (SAC) technique, which employs a *Bacillus subtilis*-derived affinity matrix (Bio-Matrix or BM) and is comparable in performance to commercial immobilized metal affinity chromatography for purifying recombinant proteins.

Sha et al. explored what mechanisms underlie the selective adhesion of probiotic *Lactiplantibacillus plantarum* HC-2 to shrimp intestine and its competitive exclusion of potential pathogens. In their genomic and proteomic study, the authors revealed that a drop in FtsH protease activity raised membrane protein levels and thereby increased the adherence of this strain to shrimp mucus. Most of the membrane proteins were associated with transport and regulation of cellular processes. Interestingly, co-culturing of the *L. plantarum* HC-2 strain with the pathogenic *Vibrio parahaemolyticus* E1 strain showed an upregulation of genes for some of these membrane proteins, thus suggesting a possible mechanism to competitively exclude pathogens from colonizing the host gut. Sha et al. contend that their findings will help advance the screening of new probiotics for maintaining intestinal health and stability.

In summary, this Research Topic has gathered an eclectic mix of insights for fully understanding the mechanisms of probiotic action and function, and thereby lays a strong theoretical foundation for future research endeavors and innovation in this field of study.

## Author contributions

JK: Writing—original draft, Writing—review & editing. VK: Writing—original draft, Writing—review & editing. IvO: Writing—original draft, Writing—review & editing.

